# Clinical symptoms, biochemistry, and liver histology during the native liver period of progressive familial intrahepatic cholestasis type 2

**DOI:** 10.1186/s13023-024-03080-6

**Published:** 2024-02-10

**Authors:** Hiroki Kondou, Satoshi Nakano, Tadahaya Mizuno, Kazuhiko Bessho, Yasuhiro Hasegawa, Atsuko Nakazawa, Ken Tanikawa, Yoshihiro Azuma, Tatsuya Okamoto, Ayano Inui, Kazuo Imagawa, Mureo Kasahara, Yoh Zen, Mitsuyoshi Suzuki, Hisamitsu Hayashi

**Affiliations:** 1 Department of Pediatrics, Kindai University Nara Hospital, Nara, Japan; 2 Department of Pediatrics, Juntendo University Graduate School of Medicine, Tokyo, Japan; 3 Laboratory of Molecular Pharmacokinetics, Graduate School of Pharmaceutical Science, The University of Tokyo, Tokyo, Japan; 4 Department of Pediatrics, Osaka University Graduate School of Medicine, Osaka, Japan; 5 Department of Clinical Research, Saitama Children’s Medical Center, Saitama, Japan; 6 Department of Diagnostic Pathology, Kurume University Hospital, Fukuoka, Japan; 7 Department of Pediatrics, Yamaguchi University Graduate School of Medicine, Yamaguchi, Japan; 8 Department of Pediatric Surgery, Kyoto University Hospital, Kyoto, Japan; 9 Department of Pediatric Hepatology and Gastroenterology, Saiseikai Yokohama City Eastern Hospital, Kanagawa, Japan; 10 Department of Pediatrics, University of Tsukuba Hospital, Ibaraki, Japan; 11 Organ Transplantation Center, National Center for Child Health and Development, Tokyo, Japan; 12 Institute of Liver Studies, King’s College Hospital and King’s College London, London, UK

**Keywords:** BSEP, Pediatric liver disease, Liver transplantation, NaPB, Ultra-rare diseases

## Abstract

**Background:**

Progressive familial intrahepatic cholestasis type 2 (PFIC2) is an ultra-rare disease caused by mutations in the *ABCB11* gene. This study aimed to understand the course of PFIC2 during the native liver period.

**Methods:**

From November 2014 to October 2015, a survey to identify PFIC2 patients was conducted in 207 hospitals registered with the Japanese Society of Pediatric Gastroenterology, Hepatology, and Nutrition. Investigators retrospectively collected clinical data at each facility in November 2018 using pre-specified forms.

**Results:**

Based on the biallelic pathogenic variants in *ABCB11* and/or no hepatic immunohistochemical detection of BSEP, 14 Japanese PFIC2 patients were enrolled at seven facilities. The median follow-up was 63.2 [47.7–123.3] months. The median age of disease onset was 2.5 [1–4] months. Twelve patients underwent living donor liver transplantation (LDLT), with a median age at LDLT of 9 [4–57] months. Two other patients received sodium 4-phenylbutyrate (NaPB) therapy and survived over 60 months with the native liver. No patients received biliary diversion. The cases that resulted in LDLT had gradually deteriorated growth retardation, biochemical tests, and liver histology since the initial visit. In the other two patients, jaundice, growth retardation, and most of the biochemical tests improved after NaPB therapy was started, but pruritus and liver fibrosis did not.

**Conclusions:**

Japanese PFIC2 patients had gradually worsening clinical findings since the initial visit, resulting in LDLT during infancy. NaPB therapy improved jaundice and growth retardation but was insufficient to treat pruritus and liver fibrosis.

## Introduction

*ABCB11* (chromosome 2q31) encodes BSEP, an ATP-binding cassette transporter responsible for the secretion of bile salts from hepatocytes to the bile canaliculi [1–3]. Biallelic mutations in the *ABCB11* gene lead to progressive familial intrahepatic cholestasis type 2 (PFIC2), an inherited autosomal recessive liver disease. Its prevalence remains unknown, but its estimated incidence varies between 1/50,000 and 1/100,000 births, with relatively higher incidences in Saudi Arabia (approximately 1:7000) [4–6]. PFIC2 develops persistent cholestasis with normal GGT levels, intractable itching, jaundice, diarrhea, and failure to thrive in the first year of life, eventually leading to cirrhosis before adulthood [2, 3, 6]. PFIC2 is also associated with an increased risk of hepatocellular carcinoma in childhood [7]. A liver biopsy of PFIC2 patients typically shows extensive giant cell transformation of hepatocytes, lobular cholestasis, lobular inflammation, and the loss of BSEP expression using immunostaining [8, 9].

Patients with PFIC2 benefit from surgical treatment, such as partial external biliary diversion (PEBD), which decreases the size of the bile acid pool by interrupting the enterohepatic circulation [6, 10]. Liver transplantation (LTx) is the ultimate therapeutic option, but there is an additional risk in patients with PFIC2 because recurrent graft failure occurs in some patients with PFIC2 [11–13]. Pharmacotherapy for PFIC2 is under development. Selective inhibitors of the ileal bile acid transporter (IBAT), which mediates luminal uptake of bile acids in enterocytes, reduce serum BA levels and improve growth and pruritus in patients with cholestatic liver disease [14, 15]. Sodium 4-phenylbutyrate (NaPB), a drug approved for treating urea cycle disorders, has a newly identified pharmacological effect that increases the expression and function of BSEP [16, 17]. This drug improves the clinical abnormalities and liver histology of PFIC2 patients who have decreased BSEP expression at the canalicular membrane of hepatocytes but maintain its transport activity [18–20].

Due to its rarity, clinical symptoms, biochemistry, and liver histology of PFIC2 during the native liver period have remained poorly characterized. A recent report from the global consortium showed the effects of genotype and surgical interventions on clinical events such as native liver survival and the occurrence of hepatocellular carcinoma [6, 10]. However, there is no clinical information on PFIC2 regarding changes over time before surgical interventions. This fact makes it difficult for clinicians to provide optimal patient care and counseling. To obtain more detailed insights into PFIC2 during the native liver period, we report physical and biochemical parameters, clinical symptoms, and liver histology of Japanese PFIC2 patients at presentation and during the course of the disease before LTx.

## Methods

This study was approved by the institutional review boards at all participating facilities and performed by the amended Declaration of Helsinki and its later amendments or comparable ethical standards (as revised in Edinburgh 2000). In addition, we obtained informed consent in written form from patients’ parents or provided opportunities for refusal to participate in this study by opt-out. The participating medical institutions disclosed the opt-out document approved by the ethics committee.

### Patients and study design

**Fig. 1:**
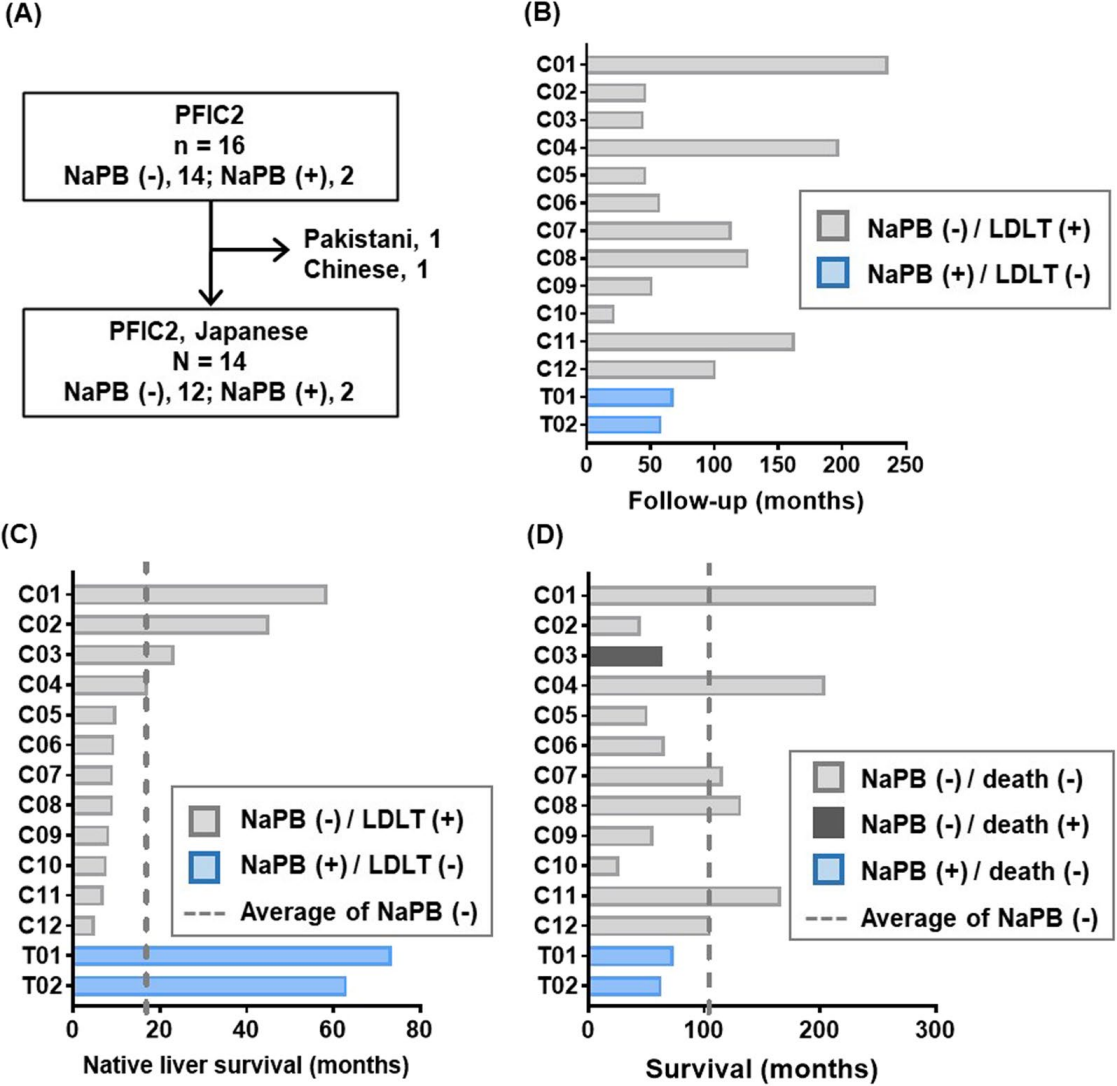
Period of follow-up, native liver survival, and survival of Japanese PFIC2 patients. **A** Flowchart of patient inclusion in this study. **B** The follow-up period of the Japanese PFIC2 patients. The patients without NaPB treatment and with NaPB treatment are shown in the gray and blue bars, respectively. **C** Native liver survival period of the Japanese PFIC2 patients. **D** Survival period of the Japanese PFIC2 patients. LDLT, living donor liver transplantation; NaPB, sodium 4-phenylbutyrate

From November 2014 to October 2015, a nationwide Japanese survey was conducted to identify patients with PFIC at facilities registered with the Japanese Society of Pediatric Gastroenterology, Hepatology, and Nutrition (207 hospitals). The clinical diagnosis of PFIC was based on unremitting hepatocellular cholestasis with intractable pruritus, jaundice with conjugated hyperbilirubinemia, and elevated serum bile acid concentrations. Complete physical examination, measurements of serological, viral, and metabolic markers, imaging, and urine screening were performed to rule out other causes of cholestasis, including hepatitis B and C virus infections, inborn errors in bile acid synthesis, and ductal origin. These patients were subjected to genetic testing for the ABCB11 gene and/or targeted next-generation sequencing panel for neonatal/infantile intrahepatic cholestasis, including *ABCB11*, to analyze all exons and flanking intron–exon boundaries of the responsible genes [21]. Patients with normal serum GGT levels who carried disease-causing mutations in both alleles of *ABCB11* and/or showed no immunohistochemical detection of BSEP in the liver were diagnosed with PFIC2. Sixteen PFIC2 patients, who consisted of 14 Japanese, 1 Chinese, and 1 Pakistani, were identified at seven facilities. Fourteen Japanese patients were enrolled in this study because of racial differences or insufficient clinical information before LTx in the two others (Fig. 1A). Investigators at each center collected demographic, clinical, and outcome data retrospectively using a pre-specified form. Data reported in the present manuscript were exported in November 2018.

### Blood chemistry test

Blood chemistry values were divided into three groups in each patient as follows. The initial and end values are defined as the values of the first visit and last visits before living donor liver transplantation (LDLT), respectively. Concerning the patients without LDLT, the values at the end of the research period were employed. The median value is defined as the median of the values between the first and last visits. To adjust for differences in reference ranges between institutions, domain experts manually standardized the dimension of each value.

### Height and weight

The height and weight values were separated into three groups in each patient in the same way as above. Then, the values were standardized based on the mean and standard deviation values calculated using the growth standard for Japanese children [22] as follows:

$${z}_{i,m}=\frac{{x}_{i,m}-{\mu }_{m}}{{\sigma }_{m}},$$

where ${z}_{i,m}$ and ${x}_{i,m}$ are the standardized and the raw values of patient $i$, and ${\mu }_{m}$ and ${\sigma }_{m}$ are the mean and standard deviation, at the corresponding month age, respectively.

### Clinical symptoms

Considering the institutional differences in diagnosis, only apparent phenotypes easily converted into binary were investigated in this study: diarrhea, grey-white stool, intracranial hemorrhage, jaundice, hepatosplenomegaly, pruritus, and rickets. All data were collected as written descriptions by clinicians and converted into the binary, whether a phenotype of interest was observed in the indicated time points. We employed three time points: the first visit (Init), the period between the first and last visits (Mid), and the last visit or the last visit before LDLT (End).

### Liver histology

Histology slides of liver biopsies and explants obtained from the PFIC2 patients were prepared and subjected to hematoxylin and eosin staining and Masson’s trichrome staining. Based on the scoring system described previously [9], three pathologists evaluated the liver histology using a multi-head microscope. The pathologists were blinded to clinical information, including the patient’s name, age, symptoms, treatment history, and serological data.

## Results

### Demographic and genetic features

Demographic and genetic information on the participants is summarized in Table 1. The number of new cases of PFIC2 each year ranged from 0 to 2. Based on this finding and the Vital Statistics published by the Ministry of Health, Labour and Welfare (https://www.mhlw.go.jp/english/database/db-hw/vs01.html), the prevalence of PFIC2 in Japan is speculated between 1/500,000 and 1/1,000,000 births. Most of the patients were girls (10/14 = 71.4%), and the median follow-up was 63.2 [47.7–123.3] months (Fig. 1B). Disease-causing mutations in both alleles of *ABCB11* were found in ten patients, 8 of whom were compound heterozygotes, and 2 were homozygous carriers. Both homozygous carriers possessed the same mutation (c.386 G > A, p.C129Y). In the remaining four patients (C02, C06, C09, and C10), disease-causing mutations in *ABCB11* were identified only in one allele. They were diagnosed with PFIC2 because immunohistological staining of BSEP in liver sections and pathogenic mutations in NR1H4 were undetectable [23]. The median age of disease onset was 2.5 [1–4] months. All patients were treated with ursodeoxycholic acid and fat-soluble vitamins but not with surgery to reduce bile salt recirculation, such as partial external biliary diversion, until the date of the last visit (T01 and T02) or LDLT (C1–C12). Two patients (T01 and T02) started NaPB therapy at 14 and 22 months of age [20]. All patients underwent LDLT (C1–C12) except for these two cases. The median age at LDLT was 9 [4–57] months, and the median from disease onset to LDLT was 7 [3–45] months. The median duration of native liver survival for the 12 patients without NaPB therapy (C1–C12) was 9.5 [8.2–18.5] months. Both patients with NaPB treatment (T01, T02) survived without LDLT for over 60 months (Fig. 1C). All but one patient (C03) are alive, and the median duration of survival of them was 69.6 [57.7–128] months (Fig. 1D). The patient (C03) died after LDLT because of antibody-induced BSEP deficiency [24].

**Table 1:** List of patients enrolled in this study

Patient number	Birth year	Gender	Diagnosis							Clinical course					
			*ABCB11* mutation 1			*ABCB11* mutation 2			IHC BSEP signal	Onset (YM)	Surgical intervention		NaPB therapy	Status	
			Exon	Nucleotide change	AA change	Exon	Nucleotide change	AA change			Surgery	Age (YM)		Outcome	Age (YM)
C01	1998	Female	14	c.1613A > G	p.Y538C	23	c.2842C > T	p.R948C	NA	1Y0M	LDLT	4Y9M	−	Alive	20Y6M
C02	2015	Male	6	c.459_462delinsA	p.I154del	ND			−	0Y4M	LDLT	3Y8M	−	Alive	3Y9M
C03 ^[24]^	2007	Male	13	c.1425 T > A	p.C475X	10	c.953_954delAA	p.K318RfsX33	NA	0Y5M	LDLT	1Y11M	−	Dead	5Y3M
C04	2002	Female	22	c.2703C > G	p.S901R	25	c.3248G > A	p.C1083Y	NA	0Y1M	LDLT	1Y5M	−	Alive	16Y10M
C05	2014	Female	15	c.1709C > T	p.A570V	28	c.3802C > T	p.R1268W	−	0Y4M	LDLT	0Y9M	−	Alive	4Y3M
C06	2013	Female	18	c.2150A > G	p.H717R	ND			−	0Y2M	LDLT	0Y9M	−	Alive	5Y5M
C07 ^[29]^	2009	Female	5	c.386G > A	p.C129Y	5	c.386G > A	p.C129Y	NA	0Y1M	LDLT	0Y9M	−	Alive	9Y7M
C08	2008	Female	22	c.2703C > G	p.S901R	25	c.3248G > A	p.C1083Y	NA	0Y5M	LDLT	0Y9M	−	Alive	10Y10M
C09	2014	Female	5	c.386G > A	p.C129Y	ND			−	0Y1M	LDLT	0Y8M	−	Alive	4Y7M
C10	2015	Female	10	c.989G > A	p.W330X	ND			−	0Y1M	LDLT	0Y7M	−	Alive	2Y2M
C11	2005	Male	5	c.386G > A	p.C129Y	15	c.1723C > T	p.R575X	NA	0Y2M	LDLT	0Y6M	−	Alive	13Y8M
C12	2010	Male	2	c.22C > T	p.R8X	18	c.2150A > G	p.H717R	−	0Y1M	LDLT	0Y4M	−	Alive	8Y8M
T01 ^[20]^	2012	Female	5	c.386G > A	p.C129Y	14	c.1460G > A	p.R487H	−	0Y4M	−	−	+	Alive	6Y1M
T02 ^[20]^	2013	Female	5	c.386G > A	p.C129Y	5	c.386G > A	p.C129Y	−	0Y3M	−	−	+	Alive	5Y2M

[20] Nakano S, et al., Sci Rep. 2019 Nov 19;9(1):17075, [24] Masahata K, et al., Transplant Proc. 2016 Nov;48(9):3156−62, [29] Imagawa K, et al., J Hum Genet. 2018 May;63(5):569–77

*M* month, *NA* not available, *ND* not detected, *Y* year

### Growth

**Fig. 2:**
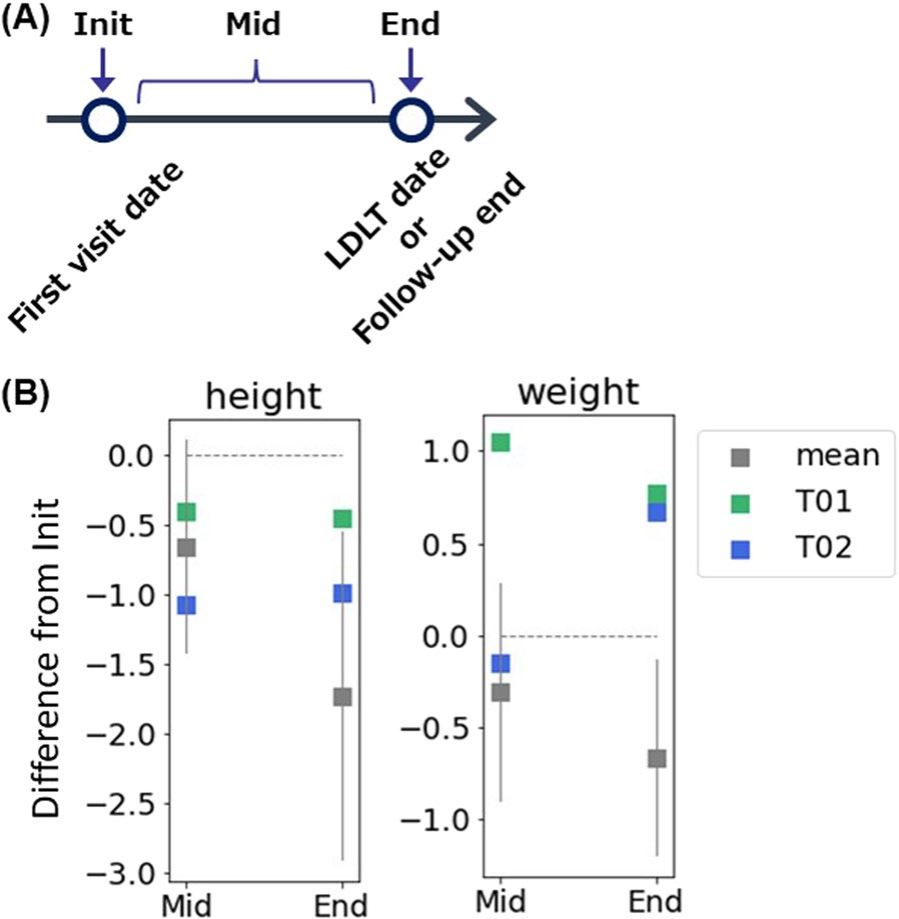
Height and weight gain in Japanese PFIC2 patients with native liver. **A** Definition of the category of the follow-up time points. Init, the initial visit; End, the last visit (T01 and T02) or the last visit before LDLT (C01, C02, C03, C06, C08, and C10); Mid, visits between the initial and the end. **B** Height and weight gain. Height and weight were scored as z score based on the mean and SD of the Japanese population at each month’s age. The difference in z-score of height and weight between Init and Mid or End was calculated and plotted. The median z-score measured during the Mid-period was used for the Mid-values. Data concerning patients without NaPB therapy are shown as means with 95% CI (n = 6; C01, C02, C03, C06, C08, and C10). A grey horizontal and dotted line indicates 0

The Z-scores for height and weight were available in 8 of the 14 patients at the first visit. Patients who did not receive NaPB therapy (C01, C02, C03, C06, C08, and C10) had progressive growth retardation in both height and weight over time. Two patients treated with NaPB therapy (T01 and T02) showed improvement in growth retardation, especially in weight, which normalized at the end of observation (Fig. 2). The Z-score for height remained unchanged in both patients.

### Clinical features

**Fig. 3:**
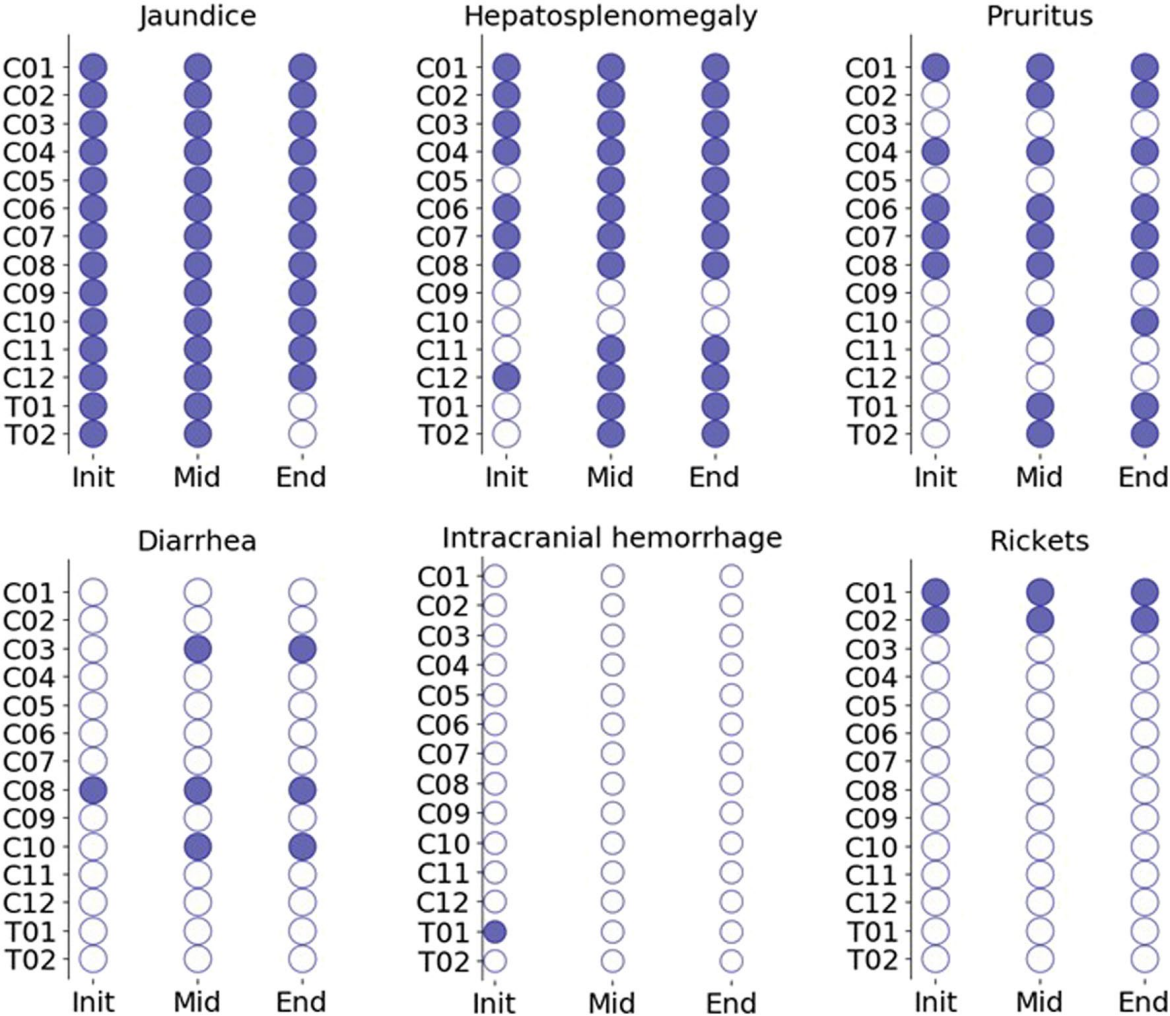
Clinical symptoms in Japanese PFIC2 patients with native liver. The closed and open circles show that each clinical symptom was observed and not observed at the indicated period, respectively. Init, the initial data available; End, the last visit (T01 and T02) or the last visit before LDLT (C01–C12); Mid, visits between the initial and the end

Changes in clinical symptoms from the initial visit are shown in Fig. 3. The most common symptom observed was jaundice (14/14 = 100%), followed by hepatosplenomegaly (12/14 = 85.7%) and pruritus (9/14 = 64.3%). Diarrhea (3/14 = 21.4%) and rickets (2/14 = 14.3%) were observed. Intracranial hemorrhage was identified in one case (T01) at the initial visit, but she recovered without neurological sequelae. In two cases (T01 and T02) where NaPB administration was started after the initial visit, jaundice improved, but pruritus remained at the end of observation.

### Laboratory evaluations

In blood biochemistry at the first visit, all patients had elevated transaminases, total and direct bilirubin (T-bil, D-bil), and total bile acids (TBA). Still, GGT was within the standard limit in all cases. Cholinesterase (ChE) was decreased in half of the patients (7/14 = 50%), and PT-INR was prolonged in 8 patients (8/13 = 61.5%). Platelet counts and coagulation function were within normal limits in T01, who presented with intracranial hemorrhage. None had hypoalbuminemia at the initial presentation.

**Fig. 4:**
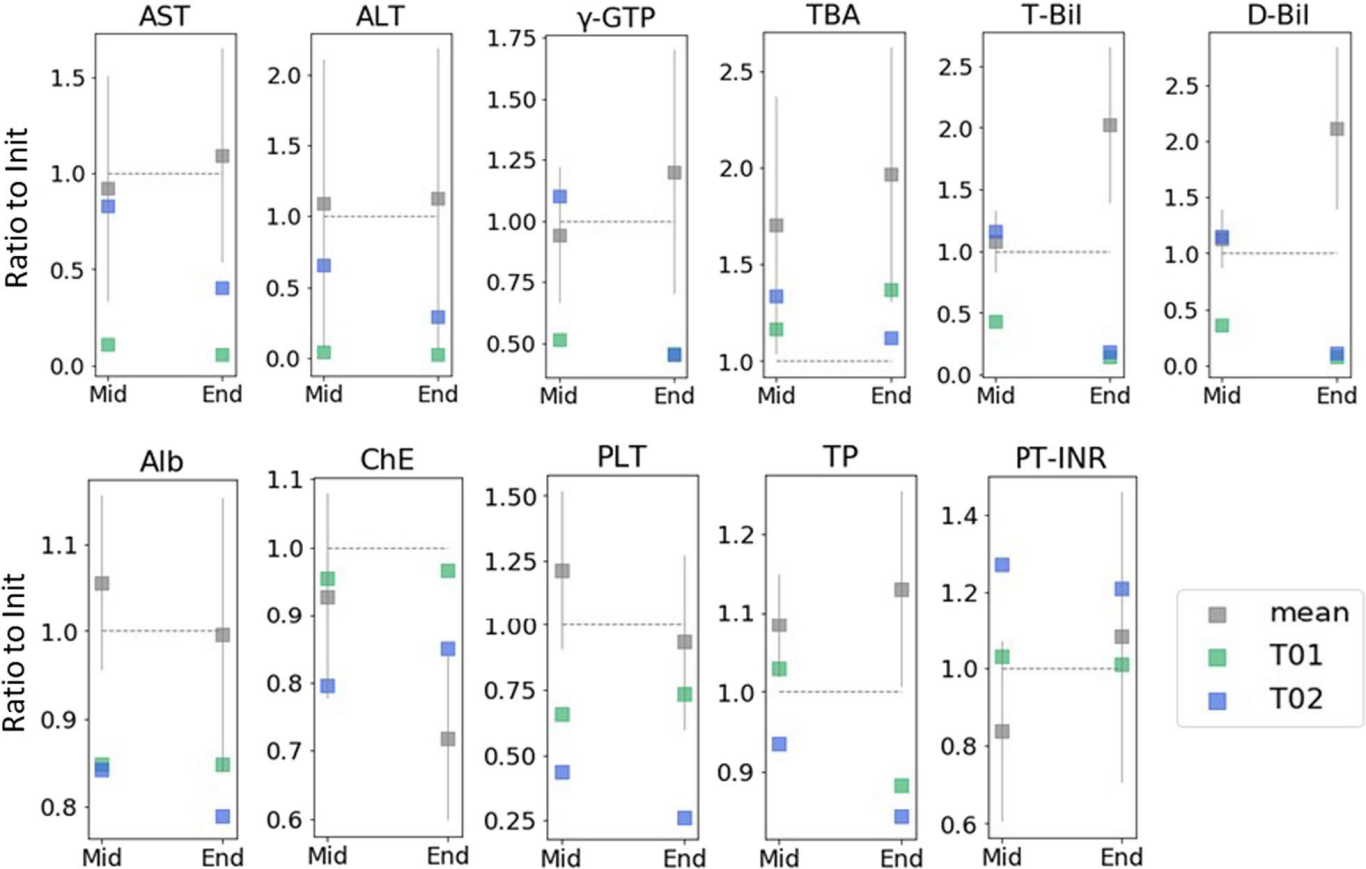
Biochemistry in Japanese PFIC2 patients with native liver. Each test result is shown as a ratio to values at the Inti. The median measured during the Mid-period was used for the Mid-values. Data concerning patients without NaPB therapy are shown as means with 95% CI (n = 12). A grey horizontal and dotted line indicates 1.0. Init, the initial visit; End, the last visit (T01 and T02) or the last visit before LDLT (C01–C12); Mid, visits between the initial and the end

Changes in biochemical blood tests after the first visit are shown in Fig. 4. Patients who resulted in LDLT (C01–C12) had progressively elevated T-bil, D-bil, and TBA levels. ChE levels and platelet counts decreased in these patients, and PT-INR was prolonged. No such changes in the values of these tests were observed in the patients with NaPB therapy (T01 and T02). In both cases, the elevated levels of transaminase and T-bil and D-bil were normalized at the end of observation. Total protein level was decreased in both patients with NaPB therapy (T01 and T02), and the opposite trend was observed in the patients who resulted in LDLT (C01–C12).

### Liver histological analysis

The liver histology of the participants is summarized in Table 2. Liver pathology specimens were available in 9 of the 14 cases, of which 6 had the specimens available at two-time points. In four cases (C01, C03, C06, and C12), the first liver specimens were collected by percutaneous needle liver biopsy, and the second liver specimens were taken from the explant liver at LDLT. Two patients who received NaPB therapy (T01 and T02) underwent percutaneous needle liver biopsy for collecting liver specimens at both time points. The median age at the first liver biopsy was 4 [4–6.5] months, and the median time from onset to the first liver biopsy was 3 [1–4] months. The collected specimens were analyzed using the previously described method [9]. In this method, the score increases as the histology worsens. The median scores of each histological finding in the initial liver biopsy were as follows: cholestasis: 3 [3–6.5] (min: 0, max: 9), parenchymal change: 5 [4.5–9] (min: 0, max: 11), portal tract change: 3 [3–4.5] (min: 0, max: 9), fibrosis: 3 [3, 4] (min: 0, max: 8). In the four cases (C01, C03, C06, and C12) who underwent LDLT and had liver specimens available at two-time points, all histological findings were worse in the explanted livers at LDLT compared to the initial liver biopsy specimens. The median scores of each histological finding at LDLT were as follows; cholestasis: 8 [6–8], parenchymal change: 10 [9–11], portal tract change: 5 [4–6], fibrosis: 5.5 [5–7]. All histological findings were improved or unchanged in 2 cases who received NaPB therapy (T01 and T02).

**Table 2:** Liver histology score

Patient number	Init						End					
	Age	Sampling	Score				Age	Sampling	Score			
			Cholestasis	Parenchymal change	Portal tract change	Fibrosis			Cholestasis	Parenchymal change	Portal tract change	Fibrosis
C01	3Y	Biopsy	2	4	3	3	4Y9M	Explant	8	9	6	7
C02	NA	NA	NA	NA	NA	NA	NA	NA	NA	NA	NA	NA
C03	0Y6M	Biopsy	3	5	3	1	1Y11M	Explant	8	11	6	7
C04	NA	NA	NA	NA	NA	NA	NA	NA	NA	NA	NA	NA
C05	NA	NA	NA	NA	NA	NA	0Y9M	Explant	8	10	4	5
C06	0Y7M	Biopsy	7	9	3	4	0Y9M	Explant	8	10	5	6
C07	NA	NA	NA	NA	NA	NA	NA	NA	NA	NA	NA	NA
C08	NA	NA	NA	NA	NA	NA	0Y9M	Explant	5	6	4	3
C09	0Y4M	Biopsy	7	9	5	7	NA	NA	NA	NA	NA	NA
C10	NA	NA	NA	NA	NA	NA	NA	NA	NA	NA	NA	NA
C11	NA	NA	NA	NA	NA	NA	NA	NA	NA	NA	NA	NA
C12	0Y4M	Biopsy	3	10	4	3	0Y4M	Explant	5	11	5	5
T01	0Y4M	Biopsy	3	1	7	4	5Y0M	Biopsy	3	1	1	4
T02	0Y3M	Biopsy	6	5	3	3	4Y0M	Biopsy	2	3	2	3

*M* month, *NA* not available, *Y* year

## Discussion

Because of its rarity, the clinical symptoms, biochemistry, and liver histology of PFIC2 during the native liver period have remained poorly characterized. Recent reports from the global consortium have provided information on native liver survival time and the incidence of hepatocellular carcinoma in PFIC2 patients [6, 10]. However, there is still no clinical data on PFIC2 regarding changes over time before surgical intervention. The present study investigated the detailed clinical information of Japanese PFIC2 patients during the native liver period. Fourteen Japanese PFIC2 patients identified through a nationwide survey in Japan were enrolled in this study. Based on their year of birth and the number of births in Japan, the incidence of PFIC2 in Japan was estimated to be 1/500,000 to 1/1,000,000 births. This is lower than in other countries, where it is estimated to be 1/50,000 to 1/100,000 births [4–6]. None of the participants had *ABCB11* mutations with a relatively favorable prognosis, p.E297G (c.890A > G) and p.D482G (c.1445A > G) [6, 25]. All patients except those treated with NaPB underwent LDLT at less than 5 years of age (Fig. 1C). The gradual deterioration of laboratory and histological findings up to the time of LDLT (Fig. 4 and Table 2) suggests that LDLT was inevitable to save each patient's life. The age of LTx in Japanese PFIC2 patients was earlier than previously reported in the global database study, in which the native liver survival rate at 5 years of age was 34% (66/192) in PFIC2 patients, excluding cases with p.E297G (c.890A > G) and p.D482G (c.1445A > G) [6, 25]. This may be because of the difference in the rate of patients who underwent biliary diversion, a surgical procedure to decrease the size of the bile acid pool by interrupting the enterohepatic circulation and to prolong native liver survival. None of the patients in this study underwent biliary diversion, while 23% (61/264) did in the report from the global consortium [6].

At the initial visit, all patients had elevated serum levels of AST, ALT, T-bil, D-bil, and TBA, suggesting hepatocellular damage and obstructive jaundice because of cholestasis. On the other hand, GGT levels were within the normal range, reflecting the characteristic of PFIC2. These biochemical results are consistent with the histological findings of cholestasis and parenchymal change on the initial liver biopsy (Table 2). After the initial visit, the patients who resulted in LDLT (C01–C12) had progressively elevated T-bil, D-bil, and TBA levels, decreased ChE levels and platelet counts, and prolonged PT-INR, suggesting decreased liver functional reserve. The significant components of serum total protein are albumin (50–60%) and immunoglobulins (20–30%) [26, 27]. In cirrhosis, the albumin/globulin ratio (A/G ratio) is decreased because of decreased albumin synthesis in the liver and increased immunoglobulin associated with chronic inflammation [28]. The patients who underwent LDLT were suggested to be approaching cirrhosis because of decreased serum albumin and unchanged serum total protein over time, thereby the gradual decrease in the A/G ratio. The histological findings of the explanted liver reflected these changes in biochemical tests because of worsening cholestasis, parenchymal change, portal tract change, and fibrosis (Table 2).

The two cases treated with NaPB therapy (T01 and T02) are those described in the previously published literature that examined the pharmacokinetics of NaPB and its efficacy and safety in the short term [20]. They had the c.386 G > A, p.C129Y mutation in the *ABCB11* gene, the most common mutation in Japanese PFIC2 patients (Table 1) [29]. This study provides information on the long-term treatment of NaPB in PFIC2. In both patients, biochemical and histological findings in cholestasis and hepatocellular damage were improved after the initiation of NaPB therapy. It was accompanied by the disappearance of jaundice and improvement of growth retardation. On the other hand, NaPB is not effective enough to ameliorate liver fibrosis once formed and itching. The effect of NaPB on pruritus has been evaluated in five PFIC2 patients in two studies [18, 19]. NaPB administration partially improved pruritus in 4 cases and completely in 1 case. Serum TBA level normalized only in one case with complete resolution of itching. In both patients in this study, serum TBA levels remained relatively high even after NaPB administration was started, suggesting that the pharmacologic effect of NaPB may not be sufficient for the complete resolution of pruritus. The action mechanism of NaPB is to increase BSEP expression on the hepatocanalicular membrane [16, 18, 20]. The difference in the extent of NaPB efficacy among PFIC2 patients may be due to the amount of functional BSEP in hepatocytes at baseline depending primarily on the type of mutation in each patient. Selective inhibitors of IBAT prevents intestinal absorption of bile acids, reduces serum TBA level, and improves pruritus in patients with cholestatic liver disease [14, 15]. The combination of NaPB and IBAT inhibitors may act synergistically on pruritus and provide more favorable clinical outcomes for PFIC2 patients than NaPB alone.

The relationship between genotype and phenotype has been intensively studied in the European PFIC2 cohorts [6, 30, 31]. The global database study, in which about 80% of cases are European, shows that PFIC2 patients with at least one p.E297G (c.890A > G) or p.D482G (c.1445A > G) mutation have more extended periods of native liver survival and lower incidence of hepatocellular carcinoma than other PFIC2 patients. In contrast, no significant differences were detectable in biochemistry at presentation [6]. Because p.E297G (c.890A > G) and p.D482G (c.1445A > G) are not found in Japanese patients, a similar genotype-based analysis is not practical in this study. This study included three carriers of p.C129Y (c.386 G > A) in 10 patients who did not receive NaPB therapy, but there were no notable differences in their clinical course. A possible ethnic difference is that cholelithiasis and hepatocellular carcinoma were not observed in the patients of this study. Both medical problems are relatively frequent in the European cohorts [6, 30, 31]. On the other hand, there are no significant ethnic differences in age of onset, clinical findings (jaundice and/or hepatomegaly) at onset, subsequent progression to pruritus and hepatosplenomegaly, and liver histological findings represented by mild to severe portal and lobular fibrosis around 1 year of age (Fig. 3, Tables 1 and 2) [30].

In conclusion, Japanese PFIC2 patients had gradually worsening clinical findings since the initial visit, resulting in LDLT during infancy. NaPB therapy improved cholestasis and jaundice in two patients, prolonged their survival time with the native liver, and may avoid LTx. In contrast, it was insufficient to treat pruritus and liver fibrosis. These clinical challenges may be overcome by starting the combination of NaPB with IBAT inhibitor, approved for treating cholestatic pruritus in children [15, 16], early in the course of the disease before liver fibrosis develops.

## Data Availability

All data generated or analyzed during this study are included in this published article.

## Abbreviations

BSEP: Bile salt export pump
GGT: γ-Glutamyltransferase
LTx: Liver transplantation
LDLT: Living donor liver transplantation
NaPB: Sodium 4-phenylbutyrate
PFIC: Progressive familial intrahepatic cholestasis
